# Synthetic biology approaches to enhance cancer immune responses

**DOI:** 10.3389/fimmu.2025.1698158

**Published:** 2025-11-27

**Authors:** Shuzhen Liu, Zhihao Zhong, Qihang Tu, Meiling Jin

**Affiliations:** Key Laboratory of Quantitative Engineering Biology, Shenzhen Institute of Synthetic Biology, Shenzhen Institutes of Advanced Technology, Chinese Academy of Sciences, Shenzhen, China

**Keywords:** synthetic biology, engineered bacteria, CAR-T cells, synthetic gene circuits, cancer immunotherapy

## Abstract

Synthetic biology is being widely applied in tumor therapy, ranging from attenuating microbial toxicity to constructing synthetic gene circuits and developing CAR-T cells, all of which are reshaping the landscape of cancer immunotherapy. In this review, we summarize recent advances in microbial-based therapeutics that leverage bacteria’s natural tropism for hypoxic tumor regions to deliver immunomodulatory payloads with high spatial precision. Parallel progress in CAR-T cell engineering has led to the development of armored and logic-gated constructs designed to overcome challenges such as antigen heterogeneity, the immunosuppressive tumor microenvironment, and T cell exhaustion. Synthetic biology further integrates these platforms via programmable genetic circuits capable of performing Boolean logic operations, ensuring therapeutic activation only in the presence of tumor-specific biomarkers. While this convergence offers the unprecedented precision, safety, and potency in reprogramming anti-tumor immunity, the clinical translation of these complex systems faces significant hurdles. Despite challenges in clinical translation-including safety concerns, immune clearance, and manufacturing complexity-the field is advancing toward multifunctional “smart” therapies, synergistic microbial-cell combinations, and personalized treatment strategies. Together, these innovations are defining a new generation of precision-engineered immunotherapies with the potential to transform the treatment of refractory malignancies.

## 1 Introduction

Microbial-based therapeutics have re-emerged as a promising modality. The natural propensity of certain bacteria to colonize hypoxic and necrotic tumor regions-observed over a century ago-has been repurposed through synthetic biology. Engineered bacteria are now designed as sophisticated, self-replicating biotherapeutic platforms (1, 2). By reprogramming attenuated or probiotic strains, researchers can enable localized delivery of diverse payloads-such as cytokines, tumor antigens, immune checkpoint blockers, and prodrug-converting enzymes-directly into the tumor niche. Simultaneously, cell therapy has been revolutionized by CAR-T cells, which achieve remarkable success in hematologic cancers (3, 4). However, their application to solid tumors remains hindered by antigen heterogeneity, the suppressive TME, and T cell exhaustion. Meanwhile, cancer immunotherapy has already reshaped the modern oncological landscape, providing durable remissions for patients with previously intractable malignancies. The success of immune checkpoint inhibitors and adoptive cell therapies like CAR-T cells underscores the power of harnessing the immune system to combat cancer. Yet, the efficacy of these approaches remains limited by significant challenges, including therapeutic resistance, immunosuppressive tumor microenvironments (TME), and the low immunogenicity of many solid tumors. These obstacles highlight the urgent need for innovative strategies that can overcome such barriers.

Synthetic biology has emerged as a transformative force in precision oncology, enabling the development of next-generation therapies capable of intelligently detecting and eradicating malignant cells. Recent advances include engineered gene circuits-sophisticated molecular devices that sense intracellular tumor signatures with high fidelity (5). By exploiting cancer specific biomarkers such as dysregulated transcription factors, oncogenic signaling pathways, and tumor-associated microRNA profiles, these circuits can trigger programmable therapeutic responses including targeted apoptosis, immune activation, or corrective gene editing. These technologies form a foundational element of advanced cell-based therapies. Through the integration of modular synthetic components-such as logic-gated promoters, protein switches, and post-transcriptional regulators-engineered “designer cells” aim to achieve unprecedented specificity in discriminating between malignant and healthy tissues, thereby minimizing off-target effects. Such circuits function as autonomous biosensors that dynamically interpret tumor microenvironment cues, executing therapeutic outputs only upon satisfaction of predefined molecular logic conditions (6). It is crucial to note, however, that the vast majority of these systems remain in preclinical development, with their stability, safety, and efficacy in heterogeneous human tumors yet to be rigorously established.

This review offers a comprehensive and critical analysis of these two complementary frontiers in cancer immunotherapy. We first systematically summarize the unique advantages of engineered bacteria as living therapeutics, detailing strategies to improve their safety, targeting, and immunomodulatory functions, while also highlighting the discrepancies between animal model data and more variable clinical trial outcomes. We then explore the evolution and design principles of CAR-T cells, highlighting innovative approaches to overcome barriers in solid tumors and counter T cell exhaustion, with a focus on the limited clinical validation of next-generation designs beyond hematologic malignancies. Finally, we discuss the convergence of these fields with synthetic biology, particularly the development of engineered genetic circuits that perform Boolean logic operations to accurately distinguish malignant from healthy cells. Throughout, we critically assess the translational readiness of these technologies, discussing not only the scientific promise but also the manufacturing scalability, regulatory pathways, and patient heterogeneity that will ultimately determine their clinical impact. By synthesizing recent advances and ongoing challenges, this review aims to chart the course toward next-generation, precision-engineered immunotherapies while providing a realistic appraisal of the hurdles that must be cleared redefine the fight against cancer.

## 2 Engineered bacteria for cancer immunotherapy

**Figure 1:**
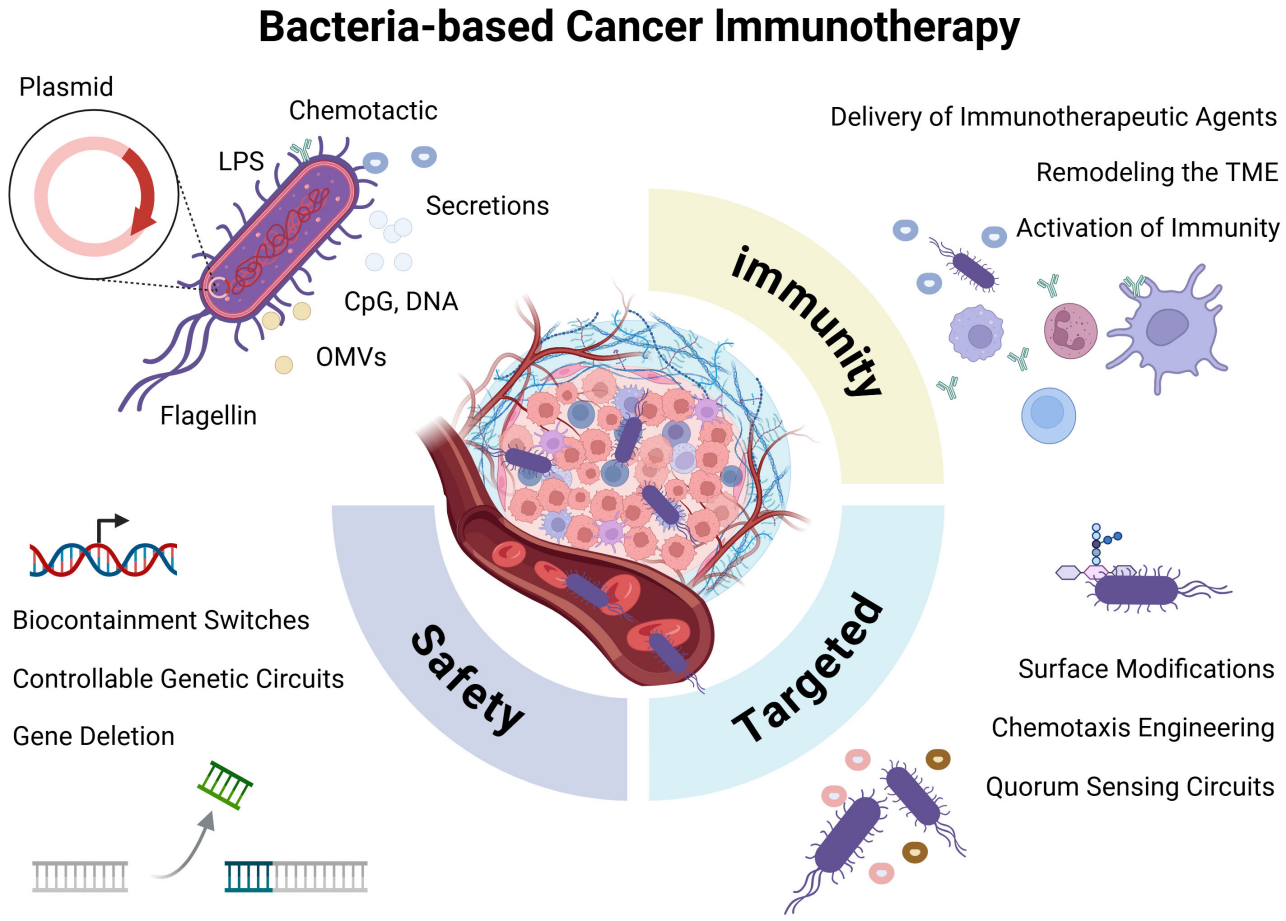
Bacteria-mediated cancer immunotherapy. Bacteria may elicit immune activation through innate structural components (e.g., LPS, flagellin, and OMVs) or via engineered payload molecules delivered via plasmid-based systems. Key engineering strategies focus on enhancing bacterial safety, improving tumor-targeting capability, and potentiating antitumor immune responses.

Cancer immunotherapy has revolutionized oncology, however, therapeutic resistance, the immunosuppressive tumor microenvironment, and low immunogenicity of solid tumors remain major obstacles. Microbe-based therapeutic strategies, particularly engineered bacterial therapies, have garnered significant attention due to their unique advantages (1, 2). Bacteria can selectively colonize and proliferate within tumors-a phenomenon observed over a century ago (7). With advances in synthetic biology, bacteria can now be precisely genetically programmed, transforming pathogenic strains into highly efficient living therapeutic platforms capable of delivering therapeutic payloads and reprogramming the tumor immune microenvironment (8, 9). This review systematically analyzes the unique advantages of engineered bacteria as cancer therapy platforms, strategies to enhance safety and targeting, immune modulation mechanisms, and the challenges, limitations, and emerging trends in clinical translation (**Figure 1**).

### 2.1 Unique advantages of engineered bacteria as cancer therapy platforms

#### 2.1.1 Intrinsic tumor targeting

Engineered bacteria possess unique advantages as cancer therapy platforms due to their intrinsic tumor-targeting capacity, immunostimulatory properties, and genetic tractability. The microenvironment of solid tumors is often characterized by hypoxia, acidity, vascular abnormalities, and immunosuppression, which collectively provide inherent targeting and colonization advantages for anaerobic or facultative anaerobic bacteria. After entering the host bloodstream, bacteria can more readily passively infiltrate and accumulate in tumor tissues due to enhanced vascular permeability, impaired lymphatic drainage, and TNF-α-induced inflammatory responses (10). The immunosuppressive nature of the tumor microenvironment-marked by reduced effector T cell activity, increased proportions of inhibitory immune cells (Tregs, MDSCs), and elevated expression of immune checkpoint molecules-further diminishes the host’s ability to clear bacterial invaders. Moreover, the abundance of metabolically accessible substrates in tumor tissues, such as lactate, various amino acids, and sugar derivatives, supplies ample nutrients to support bacterial colonization and proliferation. This multifactorial synergy enables engineered bacteria to achieve highly efficient and sustained specific targeting and colonization within tumors. For instance, Salmonella ΔppGpp strains accumulated to over 1×10¹^0^ CFU/g in tumors within three days post-administration, with a tumor-to-normal tissue ratio exceeding 10,000:1 (9).

#### 2.1.2 Potent immunostimulatory effects

Engineered bacteria can enhance antitumor efficacy through multiple mechanisms. The bacterial surface is enriched with pathogen-associated molecular patterns (PAMPs), such as LPS (lipopolysaccharide), LTA (lipoteichoic acid), peptidoglycan, flagellin, and bacterial DNA, which are recognized by pattern recognition receptors (PRRs) on innate immune cells. This recognition triggers a robust immune response, activating dendritic cells (DCs) and macrophages, and promoting the activation of cytotoxic T lymphocytes (CTLs) and natural killer (11) cells. As a result, the tumor immune microenvironment is remodeled, enhancing immune-mediated recognition and killing of tumor cells (9). Furthermore, engineered bacteria can be designed to deliver tumor neoantigens, cytokines, or toxin carriers, enabling precise therapeutic interventions (9). For instance, one strategy involves the use of engineered bacteria to express arrays of tumor neoantigens. This approach has been demonstrated in probiotic E. coli Nissle 1917 (EcN), where the expression of neoantigens promotes enhanced antigen presentation and elicits a tumor-specific immune response (12).

#### 2.1.3 Efficient living therapeutic platforms

Bacteria have a clear genetic background and mature engineering tools (such as plasmids, CRISPR-Cas systems, etc.), and their genome structures are simple and suitable for directional and precise editing. Through various promoter systems (chemical induction, thermal sensitivity, light sensitivity) and quorum sensing mechanisms, engineered bacteria can achieve metabolic pathway reconstruction, expression of exogenous anti-cancer molecules, secretion of immunomodulatory factors, and optimization of biosafety (9). For example, gene expression controlled by radiation-induced promoter RecA in Clostridium, or timed bacterial lysis and drug release using the Lux system combined with quorum sensing. At the same time, nanotechnology-assisted engineered bacteria can enhance targeting and therapeutic versatility by surface conjugation of aptamers, loading drug particles, or combining photosensitive materials (13). Compared with mammalian cells, the genome structure of bacteria is relatively simple, and it is easy to be precisely edited by plasmid vectors, phage systems, or CRISPR-Cas technology. In addition, bacteria are easy to cultivate artificially, can be rapidly expanded at low cost, short cycles, and are suitable for industrial production and standardized preparation, which lowers the economic threshold for clinical translation and provides a solid technical foundation for individualized and programmable tumor treatment (10).

### 2.2 Strategies for safety engineering

Ensuring the biosafety of engineered bacteria represents a critical prerequisite for clinical translation and *in vivo* application. Despite their promising antitumor activity, potential safety risks remain a major barrier to therapeutic use. The primary concerns include systemic toxicity (14), uncontrolled infection, excessive immune activation (15), off-target colonization (16), horizontal gene transfer (HGT) (17), and genetic instability of engineered constructs (18). These risks often originate from bacterial endotoxins such as LPS (15), which trigger excessive cytokine release, from unintended gene transfer events that disseminate resistance or virulence determinants to commensal flora, or from mutational instability of synthetic circuits, leading to unregulated gene expression or loss of biocontainment.

To mitigate these risks, multiple engineering strategies have been developed. Genetic attenuation remains the cornerstone of bacterial safety optimization, exemplified by the deletion or mutation of virulence-associated genes such as aroA, purI, or msbB, which reduce systemic toxicity and limit bacterial proliferation in normal tissues. For example, a study demonstrated that tryptophan-auxotrophic Salmonella strains engineered through this approach specifically colonized tumor sites, improving therapeutic efficacy while sparing normal tissues (19). The attenuated Salmonella strain VNP20009, which was developed based on this strategy, has entered clinical trials, but outcomes have been inconsistent due to insufficient colonization and dose-related adverse effects (20, 21). Complementary to this, strategy employs controllable genetic circuits in which therapeutic genes are placed under the regulation of tumor-specific or hypoxia-inducible promoters-such as HlyE or TFF-to restrict gene expression to the tumor microenvironment. These promoters are activated by distinctive tumor conditions, such as hypoxia, thereby minimizing off-target effects. Additionally, temperature-sensitive genetic switches that activate at 42-43°C enable spatiotemporal control of therapeutic gene expression when combined with external stimuli such as focused ultrasound (22). This method allows precise regional activation within tumors, enhancing treatment specificity and reducing systemic toxicity (22). In addition, Biocontainment switches provide another layer of safety through external signal-responsive “suicide systems.” These systems facilitate the targeted elimination of bacteria when necessary, for instance, through arabinose or tetracycline-inducible expression of lysis proteins, enabling timed bacterial clearance after treatment. Lysine-inducible systems further ensure bacterial survival only under predefined conditions, thereby offering precise control over bacterial persistence and activity in the host. Such regulatory mechanisms are particularly valuable in applications requiring tight control of bacterial behavior to ensure both safety and therapeutic effectiveness (23). Recent advances in bacterial cancer therapy have focused on optimizing delivery routes-such as local injection or encapsulation-to reduce systemic exposure and toxicity. Engineered bacteria have been combined with immune modulators, including cytokine regulators and checkpoint inhibitors, to enhance efficacy while mitigating cytokine storm risk. Notably, E. coli Nissle-derived SYNB1891 integrates a STING agonist payload with a self-lysis circuit, enabling localized immune activation and improved tumor control (24). In preclinical and phase I studies, SYNB1891 induced robust intratumoral IFN signaling with favorable safety and minimal systemic cytokine release (14). However, challenges remain in achieving consistent colonization dynamics and balancing immune potency with biosafety in complex tumor microenvironments.

### 2.3 Strategies to enhance tumor targeting

Enhancing the tumor-targeting capability of engineered bacteria not only improves treatment safety but also increases the precision and efficacy of bacteria-based cancer therapy. Chemotaxis engineering involves the overexpression or introduction of receptors sensitive to tumor-derived signals such as serine, aspartate, or hypoxia, enabling active bacterial navigation toward tumors. For example, engineered Salmonella strains with enhanced chemotaxis have been shown to achieve up to 1000-fold greater accumulation in tumors compared to normal tissues (25). Surface modifications offer another targeting mechanism, where the display of tumor-specific peptides or antibody fragments, such as anti-EGFR scFv-on the bacterial surface promotes enhanced adhesion and infiltration into tumor tissues. A 2024 study demonstrated that such modifications significantly increase bacterial accumulation in tumors and improve therapeutic outcomes (26). Additionally, quorum-sensing circuits are employed to control therapeutic protein expression only when a sufficient bacterial density is reached within the tumor, thereby minimizing premature immune clearance and ensuring effective local drug release. Advances in synthetic biology have further refined these systems, allowing precise spatiotemporal regulation of treatment activity and enhancing both efficacy and safety (8).

### 2.4 Modulating anti-tumor immune responses: from immune adjuvants to immune regulators

Enhancing the immune-modulatory capacity of engineered bacteria, while ensuring biosafety, represents a critical strategy for strengthening anti-tumor immune responses. Through multiple coordinated mechanisms-including localized delivery of immunotherapeutic agents, remodeling of the tumor immune microenvironment, and activation of innate immune signaling-engineered bacteria synergistically potentiate anti-tumor immunity, establishing themselves as versatile biotechnological platforms for cancer immunotherapy.

One major approach involves the localized delivery of immunotherapeutic agents. For instance, bacteria can be designed to express cytokines such as IL-2, GM-CSF, or IFN-γ, which activate and recruit T cells, NK cells, and dendritic cells directly within the TME, thereby minimizing systemic toxicity. Engineered Salmonella strains delivering GM-CSF have been shown to enhance the infiltration of M1 macrophages, dendritic cells, and CD8^+^ T cells in murine tumor models (27). Similarly, *in situ* production of immune checkpoint inhibitors-including anti-PD-1, anti-PD-L1, or anti-CTLA-4 scFvs-enables high local concentrations while reducing off-target effects, as demonstrated by Neospora caninum engineered to express anti-PD-L1 scFv-Fc, which effectively binds human PD-L1 and potentiates antitumor immunity. Bacterial delivery of tumor antigens or neoantigens further serves as an *in situ* vaccine by activating antigen-presenting cells and priming tumor-specific T cells, offering a promising strategy for eliciting sustained immune responses (12).

Beyond payload delivery, engineered bacteria can actively remodel the immunosuppressive TME. Strategies include the targeted depletion of regulatory immune cells such as Tregs and MDSCs through expression of neutralizing agents like anti-CD25 scFv or immunostimulatory cytokines such as IL-12. For example, IL-12-expressing Salmonella reprograms tumor-associated macrophages toward a pro-inflammatory phenotype, thereby enhancing antitumor immunity and treatment efficacy. Additionally, bacterial expression of ECM-degrading enzymes, such as hyaluronidases-facilitates immune cell infiltration by breaking down physical barriers like hyaluronic acid, improving both drug penetration and T cell access into tumor cores (28).

Furthermore, engineered bacteria activate innate immune pathways through pathogen-associated molecular patterns (PAMPs) and synthetic immunostimulants such as STING or TLR agonists. These signals promote dendritic cell and macrophage activation, enhance antigen presentation, and stimulate pro-inflammatory cytokine production. Notably, cytosolic bacteria have been shown to synergize with STING agonist therapies through TLR pathway activation, highlighting their potential in combined immunotherapeutic strategies (29).

### 2.5 Clinical translation of engineered bacteria: advances, challenges, and future directions

In recent years, engineered microbial therapies have progressively advanced from proof-of-concept studies to early-stage clinical translation in the field of cancer immunotherapy. Several clinical trials have preliminarily demonstrated their safety and feasibility while elucidating underlying immune activation mechanisms (**Table 1**). For instance, the early intravenous administration of attenuated Salmonella VNP20009 in a Phase I trial confirmed its safety and ability to colonize tumor sites, though its monotherapeutic antitumor efficacy remained limited, indicating the need for further engineering to enhance tumor selectivity and immunomodulatory potency (20). Similarly, Listeria monocytogenes-based vectors such as the CRS-207/ANZ-100 series have been shown to induce antigen presentation and innate immune activation, eliciting tumor antigen-specific T-cell responses and objective immunological or radiological responses in subsets of patients in early-phase studies (33, 34). However, subsequent randomized controlled trials failed to confirm significant efficacy, underscoring the necessity for more precise biomarker stratification or rational combination strategies to improve response rates.

**Table 1:** Landmark and recent advances in engineered bacteria for cancer therapy.

Strain	Target/payload	Engineering strategy	Tumor model/indication	Key outcome	Critical evaluation (limitations/challenges)	Phase/stage	Reference
Salmonella typhimurium VNP20009	attenuated strain: ΔpurI, ΔmsbB	Systemic i.v. administration to evaluate tumor colonization in patients	Metastatic melanoma, renal carcinoma (Phase I)	Partial tumor colonization; good tolerability; no objective regression	Limited tumor colonization; weak immunogenicity; systemic clearance remains rapid	Phase I (human)	(20)
Clostridium novyi-NT (spores)	Anaerobic germination (tumor hypoxia targeting)	Intratumoral injection of spores to induce tumor lysis	Murine, canine, and early human tumors	Strong tumor necrosis in hypoxic cores; immune activation	Necrosis control difficult; risk of local inflammation and infection; limited systemic effect	Preclinical → Early clinical	(30)
Salmonella	Therapeutic payloads (cytotoxic or immunomodulatory)	Quorum-sensing–based synchronized lysis circuit (SLC)	Murine colon and melanoma models	Pulsatile payload release improved tumor control and safety	Circuit stability & plasmid loss remain issues; inter-patient quorum variability limits clinical translation	Preclinical	(31)
E. coli Nissle 1917	Neoantigen arrays (tumor-specific epitopes)	Codon-optimized expression & APC-targeted antigen release	CT26 (colon) and B16 (melanoma) mouse models	Strong CD8^+^/CD4^+^ T-cell activation; tumor regression; long-term protection	Only preclinical; lacks validation in human immune context; potential safety concerns from gut colonization	Preclinical	(12)
E. coli Nissle 1917 (SYNB1891)	Cyclic dinucleotides (CDNs)	Tumor-responsive promoter driving CDN biosynthesis	Advanced solid tumors (Phase I trial)	STING pathway activation, immune gene upregulation, manageable safety	Limited efficacy as monotherapy; cytokine-related toxicity; delivery still intratumoral (not systemic)	Phase I	(14)
E. coli Nissle 1917	IL-2 (immunostimulatory cytokine)	Tumor-specific inducible promoter controlling IL-2 secretion	CT26 and B16 tumor-bearing mice	Increased CD8^+^ infiltration and partial tumor regression	IL-2 diffusion limited; transient cytokine levels; no systemic immune memory	Preclinical	(32)
E. coli Nissle 1917 (EcN)	STING agonists/cytokines	Tumor-inducible payload expression (SYNB1891 platform)	Mouse models and early human trials	Demonstrated modularity of probiotic therapy platform	Microbiome interactions complex; oral vs. intratumoral delivery efficacy unclear	Preclinical → Phase I	(24)

A prominent current direction involves designing engineered bacteria as “intratumoral drug factories” for localized production of immunomodulatory agents. For example, the engineered Escherichia coli Nissle 1917 strain SYNB1891, which synthesizes a STING agonist under hypoxic tumor conditions, demonstrated controllable immunogenicity and an acceptable safety profile in a Phase I trial, supporting the clinical feasibility of local innate immune activation and its potential for combination with systemic immunotherapies (14). Concurrently, combination strategies integrating engineered bacteria with immune checkpoint inhibitors, chemotherapy, or radiotherapy have shown synergistic potential. The intratumoral injection of Clostridium novyi-NT, for instance, induced localized tumor necrosis and immune activation, though its clinical application remains limited by complications such as infection and inflammatory toxicity, highlighting the critical need to balance efficacy with toxicity management (35).

Despite these advances, the clinical translation of engineered bacteria continues to face multiple challenges. Key issues include safety concerns such as unpredictable bacteremia and systemic inflammatory responses, insufficient controllability of *in vivo* colonization dynamics and transgene expression, limited reproducibility in manufacturing, and frequent failures in translation from preclinical models to human trials. Additionally, the complex regulatory landscape for live biologic products poses further obstacles (8). To address these limitations, future research should focus on developing externally controllable genetic circuits to improve the safety window; optimizing tumor-specific targeting elements to enhance selective bacterial accumulation; establishing standardized manufacturing processes and *in vivo* tracking methodologies; and identifying optimal combination schedules with existing immunotherapies alongside predictive biomarkers (36). Through multidisciplinary collaboration, engineered microbial therapies are poised to achieve broader clinical application in the era of precision cancer immunotherapy.

## 3 CAR-T cancer vaccines

Adoptive cellular immunotherapy, particularly CD19-targeted CAR-T cell therapy, has achieved remarkable success in curing numerous patients with relapsed or refractory B-cell lymphomas and leukemias, heralding a new era in cancer treatment (37). Chimeric Antigen Receptors (CARs) are synthetic receptors engineered to enable T cells to recognize tumor-associated antigens in an MHC-independent manner, thereby efficiently activating T-cell responses (38). However, the efficacy of CAR-T therapy in solid tumors remains limited, hindered by challenges such as target antigen selection, immunosuppressive tumor microenvironments, and insufficient T-cell persistence (3). This review aims to comprehensively summarize advances in CAR-T technology and highlight the latest strategies developed to overcome these critical limitations (38, 39).

### 3.1 CAR-T cell generational evolution and design principles

**Figure 2:**
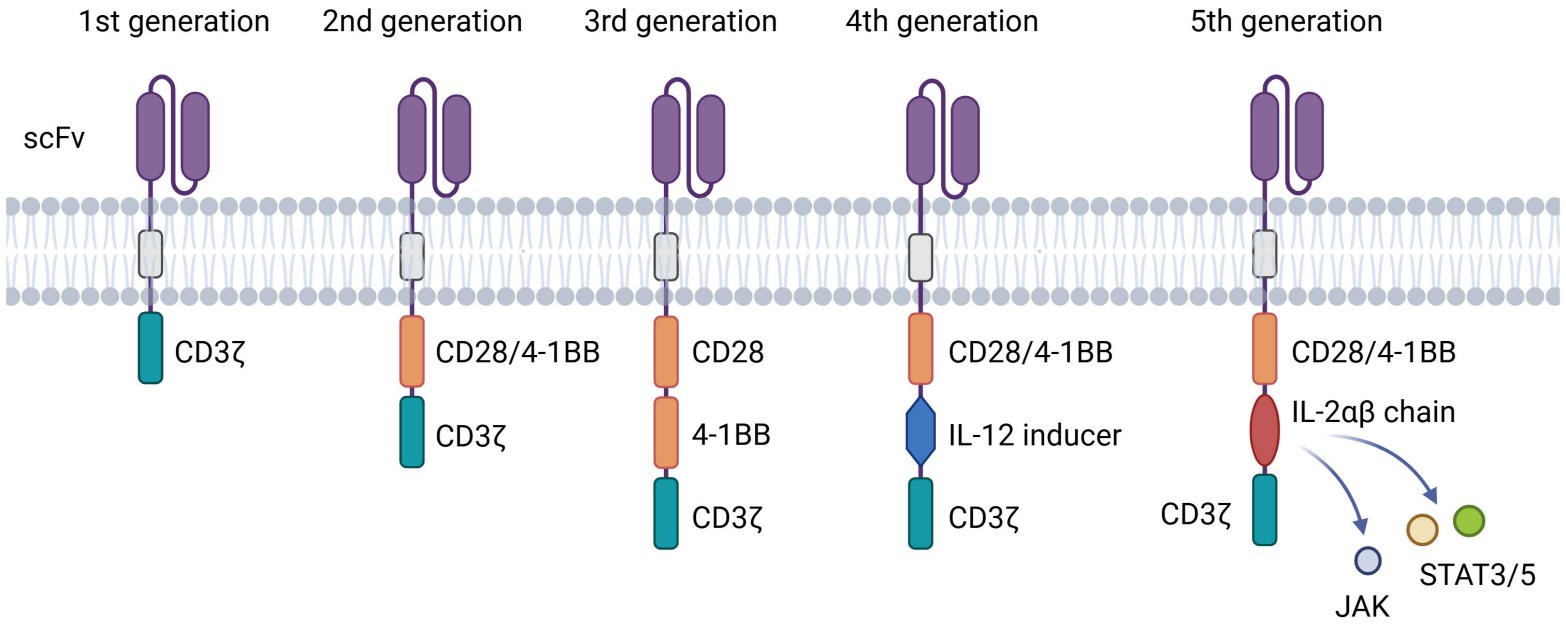
Structures of different generations of CAR. The evolution of CAR-T technology from the 1st to the 5th generation. The core evolutionary path lies in the combination and innovation of intracellular signaling domains, aiming to overcome challenges such as T-cell exhaustion, poor persistence, and tumor immune suppression.

CAR-T cell therapy has evolved through multiple generations, each refining design to enhance anti-tumor function and persistence (**Figure 2**). First-generation CARs incorporated only CD3ζ signaling, exhibiting limited expansion and efficacy (40). Second-generation constructs introduced co-stimulatory domains (e.g., CD28 or 4-1BB), markedly improving T-cell activation, persistence, and clinical outcomes-forming the basis of approved therapies like Yescarta and Kymriah (41) (42). Third-generation CARs combined two co-stimulatory domains but have not yet demonstrated clear clinical superiority (43). Fourth-generation “armored” CARs include inducible cytokine expression systems (e.g., IL-12) to modulate the tumor microenvironment and may incorporate safety switches (44). Fifth-generation designs integrate cytokine receptor domains (e.g., IL-2Rβ–STAT) to activate JAK-STAT signaling, enhancing proliferation and exhaustion resistance across hematologic and solid tumors (45). Universal platforms such as SUPRA and BBIR CARs are also emerging to enable adaptable antigen targeting (44).

Key molecular elements finely regulate CAR-T function. The scFv governs antigen specificity, where affinity must balance potency and safety to avoid on-target/off-tumor effects and T-cell exhaustion (46). The hinge region modulates binding accessibility and immune synapse formation, with length and composition (e.g., IgG4 or CD8α-derived) directly influencing efficacy (45). The transmembrane domain, often derived from CD28 or CD8α, ensures stable membrane expression and influences signaling efficiency. Finally, the intracellular signaling domains determine functional outcomes: CD3ζ provides primary activation, while co-stimulatory domains shape response kinetics-CD28 promotes rapid effector activity, and 4-1BB enhances metabolic fitness and long-term persistence (46, 47).

### 3.2 Innovative CAR designs to overcome tumor heterogeneity

Ideal tumor antigens should be highly and uniformly expressed on tumor cells while absent from normal tissues. However, the identification of such tumor-specific antigens has remained a persistent challenge. To date, in both hematologic and solid malignancies, most tumor antigens are also expressed, at least in part, on certain subsets of normal cells. Consequently, adoptive T-cell therapies targeting tumor-associated antigens (TAAs) rather than truly tumor-specific antigens carry an inherent risk of off-tumor toxicity. Clinical experience with CAR-T cell therapies has demonstrated that while effectively targeting tumors, these treatments can induce varying degrees of off-target toxicities, ranging from predictable and manageable to unforeseen and potentially fatal. To address tumor heterogeneity and antigen escape-key challenges leading to treatment failure in CAR-T therapy-innovative logic-gated and universal CAR systems have been developed. Logic-gated CARs employ Boolean computing principles to enhance specificity and adaptability. For instance, “AND-gate” CARs require simultaneous recognition of two tumor-associated antigens to trigger full T-cell activation, significantly improving tumor selectivity and reducing off-target effects (48). In contrast, “OR-gate” CARs allow activation upon engagement of either antigen, expanding target coverage and mitigating the risk of antigen-negative relapse. Another advanced logic design incorporates inhibitory CARs (iCARs), where T cells co-express an activating CAR and a suppressor CAR targeting antigens on healthy tissues. Upon binding, iCARs deliver inhibitory signals-via domains such as CTLA-4 or PD-1-to locally suppress T-cell activity and prevent on-target/off-tumor toxicity (49).

Universal or switchable CAR platforms offer an alternative strategy by decoupling antigen binding from T-cell signaling. These systems use a universal extracellular receptor (e.g., targeting synthetic tags like fluorescein or biotin) together with soluble adaptor molecules (e.g., bispecific antibodies) that bridge the CAR-T cell to tumor antigens. This configuration allows precise external control over T-cell activity through adjustable adaptor dosing, timing, and specificity, thereby improving safety and adaptability. Recent advances, such as the SUPRA CAR system, further enhance this approach by enabling split, programmable antigen recognition and multiplexed targeting capacity in a highly tunable manner (48). While this approach offers significant safety advantages and targeting flexibility, it introduces new complexities, including the pharmacokinetics and immunogenicity of the adapter molecule, and the need for precise dosing control.

### 3.3 Enhancing antitumor immunity and advances in solid tumor research

CAR-T cell therapy faces significant challenges in solid tumors due to the immunosuppressive tumor microenvironment (TME). To counter this, “armored” CAR-T cells have been engineered to secrete immunomodulatory cytokines such as IL-12, IL-15, or IL-7, or to express enhanced cytokine receptors (e.g., IL-7R), enabling them to withstand inhibitory signals, promote persistence, and recruit endogenous immune cells to foster a pro-inflammatory milieu. Additionally, CAR-T cells are being designed to directly neutralize immunosuppressive factors or cells, for instance, through expression of a TGF-β dominant-negative receptor (DNR) to block TGF-β signaling and restore T-cell cytotoxicity within the TME (11).

Advances in solid tumor targeting continue to expand the repertoire of actionable antigens, including glypican-3 (GPC3) in hepatocellular carcinoma, Claudin18.2 in gastric cancer, mesothelin (MSLN), and B7-H3 across various malignancies. Local delivery approaches such as intratumoral, intraperitoneal, or intrapleural injection-are being explored to enhance tumor-specific accumulation and minimize systemic exposure, showing promising preclinical and early clinical outcomes (50). Furthermore, combination strategies integrating CAR-T cells with immune checkpoint inhibitors, radiotherapy, chemotherapy, or targeted agents are under intensive investigation to synergistically disrupt immunosuppressive networks and amplify antitumor immunity.

### 3.4 Advances in preventing T cell exhaustion

T cell exhaustion remains a critical obstacle to the long-term efficacy and persistence of CAR-T cell therapies. To address this, multiple innovative strategies are being developed targeting transcriptional regulation, inhibitory pathways, and CAR structural design (51, 52). Transcriptional reprogramming approaches aim to suppress exhaustion-related factors such as NR4A and TOX through genetic disruption or silencing, which has been shown to enhance T cell functionality and sustain antitumor responses. Conversely, overexpression of memory-associated transcription factors like c-Jun and FOXO1 promotes a stem-like or central memory phenotype, improving proliferative capacity and exhaustion resistance (53).

Inhibition of key exhaustion pathways-such as those mediated by PD-1, TIM-3, or LAG-3-represents another major direction. CRISPR/Cas9-mediated knockout of these receptors can enhance CAR-T activity, though permanent deletion raises safety concerns regarding uncontrolled activation and autoimmunity. Alternative strategies include the use of dominant-negative receptors or transient pharmacological checkpoint inhibition to achieve more controlled modulation of immune responses (53). Next-generation CAR designs also contribute to reduced exhaustion. For instance, costimulatory domains derived from 4-1BB favor mitochondrial fitness and memory formation, leading to improved persistence compared to CD28-based constructs. Additionally, tunable CAR systems with molecular “on/off” switches allow intermittent T cell rest periods by controlling activation temporally, mitigating chronic stimulation and delaying the onset of exhaustion (54).

### 3.5 Clinical challenges and research advances-toxicity management

In recent years, chimeric antigen receptor T cell (CAR-T) therapy has achieved significant advances in cancer treatment, particularly in hematologic malignancies such as acute lymphoblastic leukemia (ALL), diffuse large B cell lymphoma (DLBCL), and multiple myeloma (MM), demonstrating high complete remission rates and favorable long-term survival outcomes (55). Representative CAR-T products, including Kymriah^®^, Yescarta^®^, and Breyanzi^®^, have received FDA approval for clinical use (56). While these results are promising, the efficacy of these therapies largely depends on single-antigen targeting, making relapse likely in patients with antigen loss or downregulation. Furthermore, high costs, complex manufacturing processes, and potential severe adverse events, such as cytokine release syndrome (CRS) and neurotoxicity, limit widespread application (**Table 2**).

**Table 2:** Representative CART for cancer therapy.

Cancer model	Target	CAR design/modification	Main outcomes	Clinical phase	Critical evaluation	Reference
Treat B-cell acute lymphoblastic leukemia (B-ALL)	CD19	2nd-generation CAR-T	High complete remission (CR) and long-term survival	FDA-approved	High efficacy, but relapse possible due to antigen loss; complex manufacturing, high cost, risk of adverse events	(56)
Treat B-cell non-Hodgkin lymphoma (NHL)	CD19	2nd-generation CAR-T	High ORR and CR	FDA-approved	Same limitations; immune escape may occur	(57)
Treat chronic lymphocytic leukemia (CLL)	CD19	2nd-generation CAR-T	High ORR and CR	FDA-approved	Same limitations; immune escape possible	(58)
Treat relapsed/refractory B-cell non-Hodgkin lymphoma (B-NHL)	CD19	3rd-generation CAR-T (CD28 + TLR2)	High ORR; no ICANS observed	Phase I	Low CRS incidence, no ICANS; efficacy and safety superior to conventional 2nd-gen CAR-T	(59)
Treat adult B-cell acute lymphoblastic leukemia (ALL)	CD19	3rd-generation CAR-T (CD28 + 4-1BB)	High ORR and CR; significantly prolonged PFS	Phase I/II	Strong efficacy, but long-term safety and cost issues remain	(60)
Treat ovarian cancer	MSLN	4th-generation CAR-T (IL-12)	Preliminary tumor reduction and survival extension	Phase I	IL-12 enhances anti-tumor activity, but may trigger systemic inflammation	(61)
Pancreatic Cancer	CEA	Hypoxia-responsive CAR-T	Reduced T-cell exhaustion; enhanced efficacy in solid tumors	Phase I/Ib	Innovative microenvironment-targeting, yet efficacy still modest; potential toxicity due to CEA expression in normal tissues	(62)

In solid tumors, CAR-T therapy faces substantial challenges. The tumor microenvironment (TME) is typically immunosuppressive, hindering CAR-T cell infiltration and function (63). Additionally, tumor antigen heterogeneity and immune escape mechanisms contribute to inconsistent therapeutic responses. Clinical data indicate that although some patients with solid tumors achieve temporary remission, overall response rates and durability remain significantly lower than in hematologic malignancies, highlighting the current limitations of CAR-T approaches in this context (64, 65).

To address these issues, recent studies have explored multi-target CAR-T designs, combination with immune checkpoint inhibitors, local delivery strategies, and gene editing optimization (66). Early-phase clinical trials show potential, but most remain at Phase I or II with small sample sizes, leaving efficacy and safety insufficiently validated. Critically, while these advanced engineering strategies theoretically enhance targeting specificity and persistence, they may increase immunogenicity, manufacturing complexity, and introduce unpredictable toxicities (38).

Future directions should adopt a cautiously optimistic perspective. Multi-target approaches and combination therapies offer strategies to mitigate antigen escape, but the persistence, safety, and TME resistance of CAR-T cells remain central bottlenecks (67). Emphasis on large-scale, multi-center randomized trials is essential to balance efficacy, accessibility, and safety. Moreover, the development of allogeneic “off-the-shelf” CAR-T products could address cost and time constraints, although long-term immune rejection risks require further investigation. 

## 4 Engineered genetic circuits for targeted recognition of intracellular tumor biomarkers

**Figure 3:**
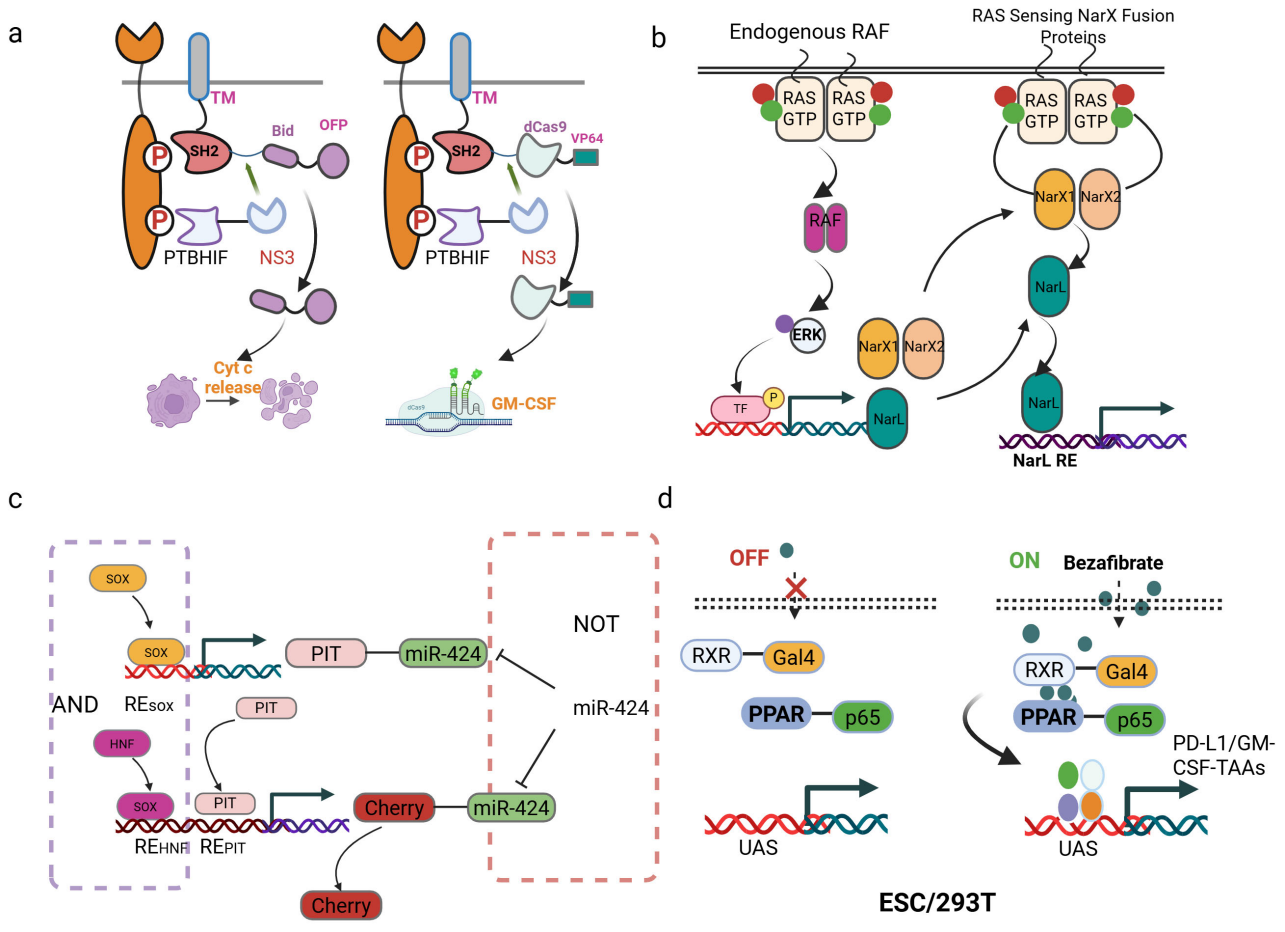
Synthetic gene circuits for cancer therapy. **(a)** Schematic of the RASER system. The system consists of two fusion proteins: one contains a membrane-anchored SH2 domain, an NS3 cleavage site, and a therapeutic payload (e.g., OFP-Bid or dCas9-VP64); the other includes a PTB domain, a HIF1α degron, and the NS3 protease. **(b)** Schematic of the RAS sensor design and mechanism. The sensor is encoded on four plasmids: two express RAS-binding domains (RBDCRD fused to either NarXN509A or NarXH399Q), a third expresses NarL, and a fourth contains an output gene (mCerulean) under a NarL-responsive promoter. **(c)** Design of a “SOX9/10 AND HNF1A/B AND (NOT miR424)” logic gate for targeted HCC therapy. **(d)** The synthetic gene circuits were regulated by bezafibrate. PPARγ and RXRα fused with potent transcriptional activators (VPR/VP64/p65 AD) and a DNA-binding domain (Gal4 DBD). Delivered via HEK293T cells, ESCs, or directly injected the plasmids containing the circuit into the tumor, the circuits reduced tumor growth.

The development of synthetic gene circuits represents a sophisticated attempt to achieve unprecedented specificity in cancer therapy (**Table 3**). One of the most promising developments is the RASER (Rewiring of Aberrant Signaling to Effector Release) system, which targets hyperactive receptor tyrosine kinases such as ErbB (6). ErbB proteins (also known as the Epidermal Growth Factor Receptor family) are receptor tyrosine kinases that serve as key regulators of cell proliferation, differentiation, and survival; their dysregulation is strongly linked to cancer development and progression (68, 69). This system consists of two engineered protein components: a membrane-tethered SH2 domain linked to a therapeutic cargo via an NS3 protease cleavage site, and a PTB domain fused to an NS3 protease under the control of a HIF1α degron. When both components colocalize at phosphorylated ErbB receptors in cancer cells, the NS3 protease cleaves its substrate to release pro-apoptotic proteins like Bid or gene-editing tools such as dCas9-VP64 (**Figure 3A**). This approach has demonstrated selective killing of ErbB-hyperactive cancer cells while sparing normal cells *in vitro*, highlighting its potential for precision oncology.

**Table 3:** Comparison of synthetic gene circuit platforms for cancer therapy.

Circuit platform	Core logic	Key inputs	Therapeutic output	Key advantages	Major challenges
RASER	AND (Co-localization	p-ErbB, Hypoxia (via HIF1α degron)	Apoptosis (Bid), Gene Activation (dCas9)	Targets aberrant signaling activity, not just overexpression	Complex multi-component delivery; potential off-target cleavage
CHOMP	AND (Dimerization)	Oncogenic RAS activation	Apoptosis (Caspase-3)	Highly modular targets “undruggable” oncogenes like RAS	Reconstitution efficiency; delivery *in vivo*
miRNAs	AND, NOT (miRNA profiles)	miR-21, -17-30a (high), miR-141,-142,-146a (low),	Apoptosis (hBax)	Exploits extensive intracellular miRNA data; high specificity.	Cell-to-cell variability in miRNA levels; delivery of the circuit.
AAV-based Multi-Input	AND, NOT (TFs & miRNA)	HNF1A/B, SOX9/10 (high), miR-424 (low	Prodrug Enzyme (HSV-TK)	Clinically relevant AAV delivery; integrates multiple input types.	Immune response to AAV; potential for insertional mutagenesis.
Transmem-brane DNA Nanomachine	AND (Membrane & Intracellular)	PTK7 (surface), miRNA-21 (intracellular)	ROS generation (Photodynamic Therapy)	Spatially-confined activation; prevents off-target effects	Complex nanomaterial synthesis; potential stability issues *in vivo*.

Similarly, the CHOMP (Circuits of Hacked Orthogonal Modular Proteases) system utilizes synthetic proteases to create logic-gated circuits that respond to oncogenic RAS activation (70). By engineering tobacco etch virus protease (TEVP) variants that reconstitute only in the presence of RAS dimerization, the system can trigger the release of activated Caspase-3, leading to apoptosis specifically in RAS-driven cancers (71). This modular design allows for the customization of protease-based circuits to target various upstream oncogenic signals, offering a versatile platform for cancer therapy.

Based on this, Senn et al. presents an innovative gene circuit strategy that integrate direct RAS sensing and transcription factor (TF) activity profiling (72). The researchers engineered a modular RAS sensor by fusing the RBDCRD domain of CRAF (a RAS effector) to engineered NarX variants. In this design, RAS-GTP binding triggers dimerization, transphosphorylation, and activation of the transcriptional regulator NarL-VP48, driving output expression (e.g., fluorescent reporters or therapeutic proteins) selectively in RAS-mutant cells (**Figure 3B**). To enhance specificity, the team incorporated MAPK pathway-responsive synthetic promoters (e.g., pFos, SRE, PY2) into an AND-gate circuit, leveraging downstream TF activation (e.g., Elk-1, c-Fos) to amplify signal discrimination between mutant and wild-type RAS. The modularity of this system allows for optimization through interchangeable components (e.g., binding domains, linkers, and transactivation domains), enabling fine-tuning for different RAS mutations (e.g., G12D) and cancer cell contexts (73). Validated across multiple RAS-driven cancer lines, the circuit demonstrates broad applicability and resistance to mutational escape, a key limitation of current RAS inhibitors (72). This work exemplifies how synthetic biology can engineer sophisticated cellular classifiers to overcome oncogenic signaling heterogeneity. However, logic-gated design is conceptually elegant and has demonstrated selective cytotoxicity *in vitro*, critical questions remain regarding their practical applicability. The multi-component nature of these protein-based circuits poses a significant delivery challenge *in vivo*. Efficient co-delivery and balanced expression of multiple engineered components in tumor cells remain major technical hurdles. Furthermore, the dependence on specific dimerization or colocalization events may be susceptible to signaling noise and heterogeneity within real tumors, potentially compromising efficacy.

The shift towards RNA-based circuits and miRNA classifiers marks an important advance in leveraging endogenous cellular machinery. The SCIP (surface T cell engager (STE), CCL21, IL-12, anti-PD1) RNA circuit and the HeLa-specific miRNA classifier demonstrate how complex Boolean logic can be implemented to enhance specificity. It introduces a modular synthetic RNA-based gene circuit platform enabling tumor-specific combinatorial immunotherapy by integrating two cancer-specific synthetic promoters (e.g., S(cMYC)p and S(E2F1)p for ovarian cancer) into an RNA-only AND gate logic (74). Stringent specificity is achieved through the circuit design: Module 1 (driven by P1) expresses a self-inhibitory transcript encoding the synthetic transcription factor GAD (GAL4-VP16), which is degraded by its intron-derived miRNA (miR1). Degradation occurs unless Module 2 (driven by P2) expresses an optimized miR1 “sponge” that competitively sequesters miR1. Only when both promoters are co-active in tumor cells does GAD stabilize and activate synthetic output promoters to express four key immunomodulators-a STE, CCL21 (75), IL-12 (76), and anti-PD1 antibody (77). *In vitro*, the circuit demonstrated cancer-specific activity, triggering selective T cell-mediated killing and IFN-γ secretion exclusively in tumor cells. *In vivo*, lentiviral delivery of the SCIP circuit in a disseminated ovarian cancer model (NSG mice + human T cells) drove significant tumor reduction, prolonged survival, and mediated potent “bystander effects”. Successful retargeting to breast cancer via promoter substitution further evidenced the platform’s modularity, highlighting its potential as a precision immunotherapy tool capable of localizing potent combinatorial immunomodulation to tumors while minimizing systemic toxicity.

A separate study built a programmable RNAi-based logic circuit that selectively identifies and eliminates HeLa cervical cancer cells by integrating endogenous microRNA expression profiles (78). Computational modeling identified a six-miRNA signature achieving specificity for HeLa versus non-HeLa cells, with experimental validation confirming Boolean logic operation: high miR-21 & high miR-17-30a & low (miR-141) & low (miR-142[3p]) & low (miR-146a). Upon target recognition, the circuit induces apoptosis via regulated expression of the pro-apoptotic protein hBax. It demonstrates high killing efficiency in HeLa cells while sparing non-target lines (e.g., HEK293) and maintaining high specificity in co-cultures. This foundational work spurred subsequent development of hybrid tumor-targeting strategies integrating tumor-specific proteins with miRNA signatures for precision cancer cell recognition and elimination, establishing a scalable platform for synthetic biology-driven oncology therapeutics. However, these systems heavily rely on the accurate identification of tumor-specific promoter or miRNA signatures-a non-trivial task given the extensive intra- and inter-tumoral heterogeneity in human cancers. A signature validated in one cancer type or cell line may not hold in another, limiting broad applicability. Moreover, the reliance on viral vectors (e.g., lentivirus, AAV) for delivery introduces its own set of challenges, including limited packaging capacity, potential immunogenicity, and the risk of insertional mutagenesis, which must be thoroughly addressed before clinical translation.

Further research applied approaches simultaneously targeting tumor-specific transcription factors and miRNAs. This study presents a clinically translatable adeno-associated virus (AAV)-based gene therapy performing multi-input biomolecular computation to achieve precise targeting and effective treatment of multifocal hepatocellular carcinoma (HCC) in mice. The authors engineered compact, modular genetic circuits implementing Boolean logic gates (AND, NOT) capable of integrating endogenous transcriptional (HNF1A/B, SOX9/10) and microRNA (miR424) inputs within individual cells (79). Packaged into systemically administered AAV vectors, this circuit specifically activated a herpes simplex virus thymidine kinase (HSV-TK) effector gene only in tumor cells exhibiting the combined molecular signature of HCC (high HNF1A/B AND high SOX9/10 AND low miR424), while minimizing off-target expression in healthy tissues. Extensive *in vitro* validation confirmed logical fidelity and selective cytotoxicity in HCC cell lines but not primary hepatocytes (**Figure 3C**). This outcome contrasted sharply with toxic effects observed when using a circuit lacking the miRNA-mediated NOT gate. This work establishes a robust, data-driven workflow-from input selection based on molecular profiling and functional validation to circuit design and *in vivo* efficacy/safety testing-for creating AAV-compatible “smart” therapeutics. It provides compelling proof-of-concept that multi-input biomolecular computation enables precise cell targeting and safe systemic delivery for next-generation cancer treatments, with potential applicability beyond HCC.

The emergence of DNA nanomachines and nanoparticle-based systems (e.g., the AuNPs/THP-ABC nanomachine and the transmembrane up conversion nanoparticle (UCNP) system) seeks to bypass biological delivery challenges by using synthetic materials. These platforms offer exciting possibilities for controlled activation and amplified signaling. The innovation centers on an “AND” logic gate requiring simultaneous cancer-specific inputs: (1) LA-apt, a DNA strand targeting membrane-overexpressed PTK-7 receptors, and (2) intracellular miRNA-21 (80). Binding of LA-apt at the cell membrane exposes a sealed miRNA-21 recognition domain while triggering receptor-mediated endocytosis of the multishell UCNP-based nanomachine, thereby confining subsequent computation intracellularly. Intracellular miRNA-21 then completes the logic operation, releasing output strand L2. Strand L2 cyclically unfolds self-quenched H012 hairpins to activate photosensitizer Rose Bengal via FRET under 808-nm NIR light, generating cytotoxic ROS for amplified photodynamic therapy (PDT). This transmembrane strategy prevents premature activation by extracellular stimuli and off-target effects on adjacent normal cells (e.g., fibroblasts, immune cells), while overcoming diffusion limitations of free DNA strands. Rigorous *in vitro* validation demonstrated cancer cell-specific activation (MCF-7, MDA-MB-231, HeLa) via ROS generation and cytotoxicity assays with minimal impact on normal cells (MCF-10a). *In vivo* studies confirmed tumor-specific nanomachine activation and significant tumor ablation in breast cancer models, alongside excellent biocompatibility and no systemic toxicity. Collectively, this work establishes a robust paradigm for cell-level precision therapy by integrating transmembrane DNA computation with up conversion-powered nanomachinery. It ensures therapeutic activation only upon sequential encounter with dual cancer biomarkers within individual cells, effectively overcoming nonspecific activation challenges in complex tumor microenvironments.

## 5 Engineered orthogonal transcription factors

Engineered orthogonal transcription factors leverage synthetic regulators derived from non-mammalian species to create minimally interfering gene circuits. These chimeric transcription factors (TFs) universally incorporate three functional domains: (1) a DNA-binding domain (DBD) targeting specific operator sequences upstream of genes of interest; (2) an actuator domain (e.g., mammalian-compatible VPR, VP16, or VP64) that recruits transcriptional machinery to promote or inhibit expression (81); and (3) a ligand-binding domain that senses input molecules. Bacterial TFs adapted for mammalian systems offer distinct advantages-including orthogonality against host networks, diverse natural ligands, modular design flexibility, and combinatorial multi-input capability. However, developing efficient mammalian gene switches requires extensive empirical optimization of binding site configurations (such as tandem repeats and spacings) to achieve acceptable signal-to-noise ratios, even with well-characterized TFs. To circumvent this bottleneck, the LOGIC platform (large orthogonal gates based on inducer-controlled cascades) exploits dimerization-dependent bacterial helix-turn-helix TFs. By fusing the dimerizing TF to either a transactivation domain (TA) or an optimized mammalian DBD (e.g., TetR or Gal4) (82), ligand-induced dimerization co-localizes TA and DBD components at target promoters. This mechanism activates transgenes without promoter retooling. This strategy enabled construction of switches responsive to vanillic acid (VA), virstatin, xylose, and gluconate, and facilitated complex logic operations.

Recent advances in synthetic biology enable the design of orthogonal gene circuits to minimize crosstalk with endogenous cellular networks while achieving precise therapeutic control. Beyond signaling pathways, synthetic biologists have developed strategies exploiting dysregulated transcription factor (TF) activity in tumors. For example, dual-promoter integrators combine two tumor-specific promoters to drive expression of split transcriptional activators, such as GAL4-DBD-Coh2 and DocS-VP16-TAD (83). Functional GAL4-VP16 transactivator assembly occurs only when both promoters are active in cancer cells, enabling Coh2-DocS interaction to induce therapeutic genes. This AND-gate logic significantly enhances targeting specificity compared to single-promoter systems.

By engineering heterologous transcription factors from bacterial systems, modular switches have been developed. The system leverages the drug-responsive heterodimerization of PPARγ and RXRα, fused to transcriptional activation domains (VPR, VP64, or p65) and the Gal4 DNA-binding domain, respectively, to activate transcription from upstream activation sequences (UAS) upon bezafibrate administration. Applied to cancer immunotherapy, this orthogonal framework was implemented in embryonic stem cells (ESCs) to deliver a dual-function circuit: bezafibrate simultaneously induces (1) tumor antigen-GM-CSF fusions to enhance immunogenicity and (2) PD-L1 nanobodies for checkpoint blockade, creating a synergistic response (84) (**Figure 3D**). *In vivo* studies demonstrated dose-dependent control, where intermediate bezafibrate concentrations activated both outputs. This resulted in an increase in CD8^+^ T cell infiltration, a reduction in PD-1^+^ exhausted T cells, and elevated Granzyme B^+^ effector populations. By combining orthogonal circuitry to isolate synthetic pathways from host interference within stem cells, this strategy establishes a clinically translatable paradigm for programmable immunotherapy. However, the development of these systems is often laborious, requiring extensive empirical optimization of binding site configuration and promoter architecture to achieve a sufficient signal-to-noise ratio. This trial-and-error process can hinder rapid iteration and deployment. While the use of small-molecule inducers like bezafibrate, as in the PPARγ/RXRα-based circuit, allows for external temporal control, it also introduces new variables of drug pharmacokinetics and patient compliance. The precise, spatially controlled delivery of the inducer to the target tissue in humans can be challenging.

In summary, while engineered genetic circuits and orthogonal transcription factors represent the cutting edge of precision oncology, their path to the clinic is paved with significant and often underappreciated challenges. The field must move beyond proof-of-concept studies in idealized models and rigorously address issues of reliable *in vivo* delivery, robustness in the face of tumor heterogeneity, and scalable manufacturing under a stringent regulatory framework.

## 6 Discussion

In this review, we have detailed the remarkable progress in engineering biological systems-from whole cells to molecular circuits-to combat cancer. From the first-generation CAR-T cells to sophisticated “armored” and logic-gated constructs, and from wild-type pathogens to precisely controlled microbial therapeutics, exemplifies the transformative power of synthetic biology and genetic engineering in medicine. The convergence of these fields is particularly compelling. Engineered bacteria and CAR-T cells, though distinct in form, share a common strategic goal: to reprogram the immunosuppressive tumor microenvironment and unleash a potent, targeted anti-tumor immune response. Bacteria act as *in situ* immune adjuvants and targeted delivery vehicles, capable of colonizing immune-excluded tumor regions and priming a response. CAR-T cells provide the lethal, antigen-specific effector arm. The future likely lies not in choosing one modality over the other, but in intelligently combining them. For instance, bacteria could be engineered to deliver chemokines or T cell engagers that recruit and enhance the function of systemically administered CAR-T cells, creating a powerful synergistic effect. However, such combinatorial approaches introduce profound complexities in safety monitoring, manufacturing, and regulatory approval that have yet to be adequately addressed. The potential for synergistic toxicity-where bacterial-induced inflammation amplifies CAR-T-related CRS-represents a particularly critical concern that requires thorough investigation.

A central theme underpinning the advances in both CAR-T and bacterial therapy is the critical importance of precision and control. The development of synthetic gene circuits, such as the RASER, CHOMP, and multi-input miRNA systems, represents a quantum leap in this endeavor. These circuits move beyond simple, always-on expression to sophisticated Boolean logic (AND, NOT, OR gates) that require the simultaneous presence of multiple tumor-specific biomarkers-such as oncogenic proteins, transcription factors, and miRNAs-to activate a therapeutic payload. This drastically enhances specificity, minimizing the risk of on-target, off-tumor toxicity that has plagued earlier therapies. The ability to spatially and temporally control therapeutic activity using external triggers like small molecules or light further adds a crucial layer of safety, enabling clinicians to modulate treatment in real time (57, 85).

Despite the exhilarating promise, the path to clinical translation is fraught with challenges. For engineered bacteria, balancing robust tumor colonization with absolute safety remains paramount. Host immune clearance, patient-to-patient variability in microbiome and immune status, and the complex manufacturing and regulatory pathways for living biologics are significant hurdles. For CAR-T cells, managing toxicities like CRS and ICANS, overcoming the suppressive solid TME, and reducing the cost and complexity of manufacturing are central concerns. The vision of “off-the-shelf” universal CAR-T products is within reach but requires solving issues of persistence and host rejection (57, 85).

The trajectory of the field points towards ever-greater integration and intelligence. We are moving towards: Multi-functional “smart” therapeutics that autonomously sense their environment, diagnose disease states, and execute appropriate therapeutic responses. Advanced combination regimens that synergize engineered cells, microbes, and conventional therapies like radiotherapy and checkpoint inhibitors. Increased personalization, with therapies tailored not just to a cancer type, but to the unique molecular profile of an individual’s tumor. Reduced costs and improved accessibility through automated manufacturing, allogeneic “off-the-shelf” platforms, and more efficient delivery systems (57, 85).

Looking ahead, the field must navigate a critical transition from proof-of-concept demonstrations to clinically viable solutions. Several key priorities emerge from our analysis: First, developing more robust and clinically tractable systems that prioritize reliability and safety over sheer complexity, with enhanced biocontainment strategies for living therapeutics. Second, conducting rigorous validation in immunocompetent models and heterogeneous tumor systems that better recapitulate human disease biology and therapeutic barriers. Third, establishing scalable, cost-effective manufacturing processes and clear regulatory pathways for these complex therapeutic modalities, particularly for combinatorial approaches. Finally, maintaining a critical focus on target antigen selection and biomarker identification grounded in comprehensive human tumor biology rather than idealized model systems. The impressive pace of technological innovation must be matched by increased attention to practical implementation. While synthetic biology has undeniably expanded the cancer therapeutic toolkit, translating sophisticated designs into effective, approved treatments requires clear-eyed acknowledgment of the persistent biological, manufacturing, and regulatory hurdles. Given the substantial resources required for clinical development, careful prioritization of the most promising approaches is essential—rather than pursuing maximum complexity for its own sake.

In conclusion, the era of engineering biology for cancer therapy shows significant promise but remains in its early stages. The field’s future impact will depend not only on continued scientific innovation but also on a disciplined focus on translational feasibility, manufacturing scalability, and clinical practicality. Through collaborative efforts that bridge basic science, clinical medicine, and regulatory science, the most promising of these technologies may eventually deliver meaningful benefit to patients with refractory malignancies
